# Investigating the biological properties of carbohydrate derived fulvic acid (CHD-FA) as a potential novel therapy for the management of oral biofilm infections

**DOI:** 10.1186/1472-6831-13-47

**Published:** 2013-09-24

**Authors:** Leighann Sherry, Emma Millhouse, David F Lappin, Colin Murray, Shauna Culshaw, Christopher J Nile, Gordon Ramage

**Affiliations:** 1 Infection and Immunity Research Group, Glasgow Dental School, School of Medicine, College of Medical, Veterinary and Life Sciences, University of Glasgow, 378 Sauchiehall Street, Glasgow G2 3JZ, UK

**Keywords:** Fulvic acid, Chlorhexidine, Biofilm, Antibacterial, Periodontitis, Inflammation

## Abstract

**Background:**

A number of oral diseases, including periodontitis, derive from microbial biofilms and are associated with increased antimicrobial resistance. Despite the widespread use of mouthwashes being used as adjunctive measures to control these biofilms, their prolonged use is not recommended due to various side effects. Therefore, alternative broad-spectrum antimicrobials that minimise these effects are highly sought after. Carbohydrate derived fulvic acid (CHD-FA) is an organic acid which has previously demonstrated to be microbiocidal against *Candida albicans* biofilms, therefore, the aims of this study were to evaluate the antibacterial activity of CHD-FA against orally derived biofilms and to investigate adjunctive biological effects.

**Methods:**

Minimum inhibitory concentrations were evaluated for CHD-FA and chlorhexidine (CHX) against a range of oral bacteria using standardised microdilution testing for planktonic and sessile. Scanning electron microscopy was also employed to visualise changes in oral biofilms after antimicrobial treatment. Cytotoxicity of these compounds was assessed against oral epithelial cells, and the effect of CHD-FA on host inflammatory markers was assessed by measuring mRNA and protein expression.

**Results:**

CHD-FA was highly active against all of the oral bacteria tested, including *Porphyromonas gingivalis*, with a sessile minimum inhibitory concentration of 0.5%. This concentration was shown to kill multi-species biofilms by approximately 90%, levels comparable to that of chlorhexidine (CHX). In a mammalian cell culture model, pretreatment of epithelial cells with buffered CHD-FA was shown to significantly down-regulate key inflammatory mediators, including interleukin-8 (IL-8), after stimulation with a multi-species biofilm.

**Conclusions:**

Overall, CHD-FA was shown to possess broad-spectrum antibacterial activity, with a supplementary function of being able to down-regulate inflammation. These properties offer an attractive spectrum of function from a naturally derived compound, which could be used as an alternative topical treatment strategy for oral biofilm diseases. Further studies *in vitro* and *in vivo* are required to determine the precise mechanism by which CHD-FA modulates the host immune response.

## Background

Dental caries, gingivitis and periodontitis are the most common microbial diseases of the oral cavity, with the majority associated with a polymicrobial biofilm [1,2]. Biofilms are a collection of multicellular microorganisms attached to one another or upon a surface, and are embedded by a protective layer of extracellular matrix (ECM) [3]. Biofilms are of greater clinical importance than their free-floating planktonic counterparts because of their innate ability to resist antimicrobial therapy and host defences. This is due to the extensive ECM production and other factors such as increased extrusion of antimicrobials through enhanced efflux pump activity [3,4].

Antimicrobial mouthwashes are one of the main therapeutic and preventative strategies currently used in the management of oral biofilm diseases, of which chlorhexidine (CHX) is widely accepted as the ‘gold standard’ [5]. This antiseptic agent has superior activity to its comparators, and is both cidal and static against microorganisms present in oral biofilms with roles in the pathogenesis of oral disease. Moreover, its substantivity provides prolonged activity through its ability to adsorb onto the pellicle found on enamel surfaces of teeth [6]. Despite this, various studies have shown long-term use of CHX may not be practical as it is associated with staining of the teeth and taste alterations [7,8]. Furthermore, recent reports of adverse events, including anaphylactic reactions, to this compound have been described [9]. It has also been shown recently to be ineffective against biofilms grown from clinical isolates [10].

The prevention and treatment of oral biofilm diseases, such as periodontal diseases and mucosal infections, may benefit from a compound that has the potency of CHX with minimal side effects, but also elicits adjunctive biological properties, such as alteration of inflammatory pathways, which are clearly important in the pathogenesis of oral biofilm disease [11,12]. A previous study has shown CHX is able to down-regulate inflammatory mediators when challenged with a bacterial stimuli [13], though toxicological aspects of CHX may be the reason for the decreased expression. We have also shown the benefit of using natural agents in the management of oral infections, where tea tree oil (TTO) was not only non-toxic, but was able to dampen the host immune response to a fungal stimulus [14]. Furthermore, our group have previously assessed the antiseptic activity of carbohydrate-derived fulvic acid (CHD-FA), where it was shown that the compound was equally effective against *Candida albicans* planktonic and biofilm cells. Mechanistically this was identified as a membrane disruption process that was not impacted by defined biofilm adaptive resistance mechanisms [15]. CHD-FA is a colloidal organic acid, which is a major constituent of humic acids. A purified form of CHD-FA has recently been produced by a patented process, which has been shown to be non-toxic in a rat wound model, with suggestions of anti-inflammatory activity [16]. Moreover, a recent randomized, double blind, controlled trial indicated that it was well-tolerated in a clinical study of eczema [17].

The purpose of this study was to investigate whether CHD-FA has a broad-spectrum of activity against microbial biofilms of oral relevance to determine whether it could be used as an alternative to CHX based mouthwashes, which have known side effects from prolonged use. The secondary aim of the study was to determine whether the antibacterial concentration of CHD-FA had any adjunctive immunomodulatory properties, as reported elsewhere [16]. We report that CHD-FA displays rapid microbiocidal activity against orally relevant biofilms, and that it is also able to down-regulate the expression of pro-inflammatory molecules in orally relevant epithelial cells.

## Methods

### Culture conditions and standardisation

A selection of laboratory strains of commensal and pathogenic bacteria associated with oral biofilms disease were used in this study, including *Porphyromonas gingivalis* ATCC 33277 and *Fusobacterium nucleatum* ATCC 10596, which were maintained at 37°C on fastidious anaerobic agar (FAA [Lab M, Lancashire, UK]) under anaerobic conditions (85% N_2_, 10% CO_2_ and 5% H_2_, [Don Whitley Scientific Limited, Shipley, UK]). *Streptococcus mutans* 10449, *Streptococcus mitis* NCTC 12261, *Aggregatibacter actinomycetemcomitans* OSM 1123 and *Enterococcus faecalis* NCTC 5957 were grown and maintained at 37°C on Colombia blood agar (CBA [Oxoid, Hampshire, UK] in 5% CO_2_. All isolates were stored indefinitely in Microbank® vials (Pro-Lab Diagnostics, Cheshire, UK) at −80°C.

*P. gingivalis* and *F. nucleatum* were propagated in 10 ml Schaedler’s anaerobic broth (Oxoid), *S. mitis* and *A. actinomycetemcomitans* were grown in 10 ml Tryptic Soy Broth (TSB [Sigma-Aldrich, Dorset, UK]) supplemented with 0.6% yeast extract and 0.8% glucose. *E. faecalis* was grown in TSB with 0.25% glucose, and *S. mutans* was grown in 10 ml brain heart infusion (BHI [Sigma-Aldrich]), all at 37°C and at appropriate atmospheric conditions. Overnight cultures were washed by centrifugation (1000 x*g*) and resuspended in 10 ml PBS. All bacteria were then standardised and adjusted to a final working concentration of 5 × 10^4^ and 1 × 10^7^ cells/ml for planktonic and sessile susceptibility testing, respectively.

### Antibacterial susceptibility testing of planktonic and biofilm cells

During the course of this study two active compounds from the oral hygiene products Dentracine (Fulhold Ltd, Cape Town, South Africa) and Corsodyl (GlaxoSmithKline Consumer Health Care, UK) were tested, namely CHD-FA and CHX, respectively.

Antimicrobial testing to determine minimum inhibitory concentrations (MICs) of planktonic cells (PMIC) was performed using the CLSI M11-A8 broth microdilution method for anaerobic bacteria [18] and CLSI M7-A9 for bacteria grown in 5% CO_2_[19]. Minimum bactericidal concentrations (MBC) were also determined by standard plating methods.

For biofilm testing standardised *P. gingivalis*, *F. nucleatum, S. mitis* and *A. actinomycetemcomitans* were grown for 72 h and *E. faecalis* for 24 h in their respective media and atmospheric conditions, with the exception of *S. mutans* which was grown in BHI supplemented with 2% sucrose for 48 h. Biofilms were grown statically in commercially available 96-well flat bottomed microtitre plates (Corning Incorporated, NY, USA) and sessile susceptibility testing was performed as described elsewhere [20]. Following antimicrobial treatment, biofilms were washed twice with PBS and 10% alamarBlue® (Invitrogen, Paisley, UK) was added to the biofilms prior to incubation for 4 h in the dark [21]. Sessile minimum inhibitory concentrations (SMICs) were read visually and no change in colour was defined as the SMIC. Testing of all planktonic and sessile isolates was performed in quadruplicate on two separate occasions.

### Antibacterial susceptibility testing of a multi-species periodontal biofilm

A multi-species periodontal biofilm model consisting of *P. gingivalis*, *F. nucleatum, S. mitis* and *A. actinomycetemcomitans* was developed for antimicrobial testing. All bacterial species were standardised to 1 × 10^7^ cfu/mL in artificial saliva (AS) as previously described [22]. This was comprised of porcine stomach mucins (0.25% w/v), sodium chloride (0.35 w/v), potassium chloride (0.02 w/v), calcium chloride dihydrate (0.02 w/v), yeast extract (0.2 w/v), lab lemco powder (0.1 w/v), proteose peptone (0.5 w/v) in ddH_2_O. Urea was diluted in PBS (40% w/v) and added to a final concentration of 0.05% (v/v) in AS. Biofilms were prepared in 24 well plates (Corning, NY, USA) containing customised Thermanox™ coverslips (13 mm diameter, Fisher Scientific). For the addition of each bacterial species to the biofilm a standardised bacterial suspension was prepared in 500 μL of AS. Initially, *S. mitis* biofilms were grown for 24 h. Media was then removed and standardised *F. nucleatum* added, which was incubated anaerobically for a further 24 h. The supernatant was again removed and standardised *P. gingivalis* and *A. actinomycetemcomitans* in AS added to the biofilm. This was then incubated at 37°C in an anaerobic chamber for a further 4 days; each day supernatants were replaced with fresh AS. As CHD-FA was shown to be active at 0.5% v/v against all bacterial biofilms tested in this study, this concentration was used in addition to 0.2% v/v CHX to treat multispecies biofilms for 30 min, before carefully washed with PBS, and biofilm viability determined using alamarBlue®. The absorbance was read at 570 nm and the reference wavelength at 600 nm. The percentage reduction in biofilm viability was calculated according to the manufacturer’s instructions. This study was performed on three separate occasions in triplicate.

Following the antimicrobial treatment, biofilms were retained and used to quantify the number of each bacterial species found after CHD-FA and CHX treatment compared to the untreated control. Briefly, biofilms were sonicated in 1 mL of PBS for 10 min and DNA extracted using the MasterPure Gram Positive DNA Purificiation Kit (Epicentre®, Cambridge, UK), following manufacturers instructions. 1 μL of extracted DNA was added to a mastermix containing 12.5 μL SYBR® GreenER™, 9.5 μL UV-treated RNase-free water and 1 μL of 10 μM forward/reverse primers for each bacterial species. The primers used were as follows:

*A. actinomycetemcomitans* F – 5′GAACCTTACCTACTCTTGACATCCGAA3′, *A. actinomycetemcomitans* R – 5′TGCAGCACCTGTCTCAAAGC3′, *F. nucleatum* F – 5′GGATTTATTGGGCGTAAAGC3′, *F. nucleatum* R – 5′GGCATTCCTACAAATATCTACGAA3′, *P. gingivalis* F – 5′GCGCTCAACGTTCAGCC3′, *P. gingivalis* R – 5′CACGAATTCGCCTGC3′, *S. mitis* F – 5′GATACATAGCCGACCTGAG3′, *S. mitis* R – 5′CCATTGCCGAAGATTCC3′.

Three independent replicates from each parameter were analysed in triplicate using MxProP Quantitative PCR machine and MxProP 3000 software (Stratagene, Amsterdam, Netherlands). Samples were quantified based upon a previously established standard curve made up of known bacterial counts.

### Ultrastructural changes of bacterial biofilms

Scanning electron microscopy (SEM) was performed on *S. mutans*, *E. faecalis*, and the multispecies biofilms. Cells were standardised in appropriate media, as described above, and grown directly onto Thermanox™ coverslips (Nunc, Roskilde, Denmark) to allow biofilm formation. Following maturation biofilms were carefully washed with PBS before their respective treatments. Biofilms were then carefully washed twice with PBS and then fixed in 2% para-formaldehyde, 2% gluteraldehyde and 0.15 M sodium cacodylate, and 0.15% w/v Alcian Blue, pH 7.4, and prepared for SEM as previously described [23]. The specimens were sputter-coated with gold and viewed under a JEOL JSM-6400 scanning electron microscope. Images were assembled using Photoshop software (Adobe, San Jose, CA, USA).

### Toxicity of CHD-FA upon an oral epithelial cell line

OKF6/TERT2 cells (gifted by the Rheinwald laboratory, Brigham and Woman’s Hospital, Boston, USA), an immortalised human oral keratinocyte cell line, were used for determining the cytotoxicity of CHD-FA. Cells were grown to 90% confluence in keratinocyte serum-free medium (KSFM) at 37°C in 5% CO_2_ and seeded at a density of 1 × 10^5^ cells/ml in a 24 well plate. Once the cells reached 80-90% confluence, the cells were carefully washed with PBS before treatment with 0.5% (v/v) CHD-FA at the native pH 2.0 and a neutral pH of 7.0 and 0.2% (v/v) CHX for 30 min. After 30 min, the compounds were removed and the cells carefully washed with PBS to remove any residual actives. Cells were incubated in KSFM for 4 and 24 h before cellular viability was assessed using the alamarBlue® assay, as described above. Viability studies were carried out in triplicate, on three separate occasions.

### Assessing immunomodulatory properties of CHD-FA

OKF6/TERT2 cells were grown to 90% confluence in 24 well plates in defined-KSFM then pre-treated with 0.5% CHD-FA (pH 7.0) for 30 min. CHD-FA at pH 2.0 was toxic against the cell line used in this study and therefore could not allow us to analyse any potential immunomodulatory properties of this compound. Therefore, CHD-FA buffered to pH 7.0 was used to assess any further biological properties of the compound. 0.5% CHD-FA at pH 2.0 and 0.2% CHX were shown to be toxic to epithelial cells, so were not further investigated. Cells were washed with PBS to remove residual CHD-FA. As an inflammatory agonist we used the multispecies periodontal biofilm, as described above, which was attached to the underside of a hanging cell culture insert (Millipore, Massachusetts, USA) using Vaseline®, then laid adjacent to the cell monolayer. The cells were incubated with the periodontal biofilm for 4 and 24 h at 37°C in 5% CO_2_. Cells not pre-treated with CHD-FA, or not challenged with biofilms, served as appropriate controls. Following stimulation, supernatants and cell lysates were retained to assess the regulation of a panel of pro-inflammatory mediators.

Initial gene expression analysis was carried out using a custom designed RT^2^ Profiler PCR Array (Qiagen, Crawley, UK). RT^2^ Profiler arrays are a SYBR® GreenER™ based real-time PCR that allow for the detection of several genes of interest, simultaneously. Briefly, 24 μl of a mastermix containing SYBR® GreenER™, cDNA synthesised using the RT^2^ First Strand kit (Qiagen) and RNase-free water was added to each well of the RT^2^ Profiler plate, which already contained the forward and reverse primers for the genes of interest (IL-1α, IL-1β, IL-6, TNF, CSF2, CSF3, IL-8, CXCL1, CXCL3, CXCL5, CCL1 and GAPDH). Two replicates of each condition were used in the RT^2^ Profiler, which was carried out on two separate occasions.

IL-8 gene expression was analysed using SYBR® Green based qPCR (Invitrogen), using GAPDH as a housekeeping gene. The primers used were as follows: IL-8 F 5′CAGAGACAGCAGAGCACACAA3′, IL-8 R 5′TTAGCACTCCTTGGCAAAAC3′, GAPDH F 5′CAAGGCTGAGAACGGGAAG3′, GAPDH R 5′GGTGGTGAAGACGCCAGT3′. Briefly, RNA was extracted from cell lysates (Qiagen, Crawley, UK) and 55 ng/μl of cDNA synthesised using the RT^2^ First Strand cDNA synthesis kit (Qiagen, Crawley, UK), as per manufacturers instructions. 1 μl of synthesised cDNA was added to a mastermix containing 12.5 μl SYBR® GreenER™, 10.5 μl UV-treated RNase-free water and 0.5 μl of forward/reverse primers. Three independent replicates from each parameter were analysed in duplicate using MxProP Quantitative PCR machine and MxProP 3000 software (Stratagene, Amsterdam, Netherlands) and gene expression normalised to the housekeeping gene GAPDH according to the 2^*-ΔΔCT*^ method [24].

Interleukin 8 (IL-8) release into cell culture supernatants was assessed by ELISA (Invitrogen, Paisley, UK), as per manufacturer’s instructions. Results were calculated using a 4-parameter curve fit, quantifying colometric changes at 630 nm (BMG-Labtech, Ortenberg, Germany).

### Statistical analysis

Graph production, data distribution and statistical analysis were performed using GraphPad Prism (version 4; La Jolla, CA, USA). After assessing whether data conformed to a normal distribution by before and after data transforms, One-way Analysis of Variance (ANOVA) and t tests were used to investigate significant differences between independent groups of data that approximated to a Gaussian distribution. A Bonferroni correction was applied to the p value to account for multiple comparisons of the data. Non-parametric data was analysed using the Mann–Whitney *U*-test to assess differences between two independent sample groups. Student t-tests were used to measure statistical differences between the *Δ*Ct values of the two independent groups assessed in gene expression studies, although data may be represented as percentage or fold change in the figures. Statistical significance was achieved if *P < 0.05*.

## Results

### CHD-FA has rapid and broad-spectrum antibacterial activity

Corsodyl® (0.2% CHX) and Dentracine (0.8% CHD-FA) are oral formulations containing the active ingredients CHX and CHD-FA, respectively. Both agents were shown to be highly active against all planktonic and sessile oral bacteria tested (data not shown). The studies described in this manuscript focussed on the active ingredients CHX and CHD-FA and both were shown to be highly efficient at inhibiting and killing all oral isolates tested when grown either planktonically and as biofilms (Table 1). PMICs for the oral isolates ranged from 0.0625% to 0.25% for CHD-FA and from <0.00039% to 0.00078% for CHX. The PMBC/PMIC ratio for CHD-FA and CHX were ≤4, indicating both compounds displayed bactericidal activity. None of the bacterial species tested were notably more sensitive or resistant to either of the compounds. Both CHD-FA and CHX showed activity against mature biofilms, with SMICs of 0.5% for CHD-FA and from 0.003% to 0.025% for CHX. Interestingly, although CHX was effective at lower concentrations, the fold change from PMIC to SMIC ranged from only 2 to 8 for CHD-FA, whereas for CHX this ranged from 2 to 64. Overall, *P. gingivalis* was the most susceptible organism to the antimicrobial therapies tested, particularly for planktonic cells. In addition, all bacterial biofilms were equally susceptible to CHD-FA with a SMIC of 0.5%.

**Table 1:** Susceptibility profile of clinically relevant oral bacteria to four antimicrobial agents

**MIC (%)**								
	**CHD-FA**				**CHX**			
**Organism**	**PMIC**	**MBC**	**SMIC**	**Fold change**	**PMIC**	**MBC**	**SMIC**	**Fold change**
				**(SMIC/PMIC)**				**(SMIC/PMIC)**
***A. a*** *	0.25	0.25	0.5	2	0.00078	0.00313	0.025	32
***S. mitis***	0.0625	0.125	0.5	8	0.00078	0.00156	0.0125	16
***S. mutans***	0.25	0.25	0.5	2	0.00039	0.00078	0.025	64
***E. faecalis***	0.125	0.25	0.5	4	0.00156	0.025	0.003	2
***F. nucleatum***	0.125	0.5	0.5	4	0.00039	0.00078	0.195	32
***P. gingivalis***	0.0625	0.5	0.5	8	≤0.00039	0.00078	0.0625	≥16

*A. actinomycetemcomitans.

CHD-FA - (Fulhold Ltd, South Africa), CHX - (GlaxoSmithKline Consumer Health Care, UK).

**Figure 1:**
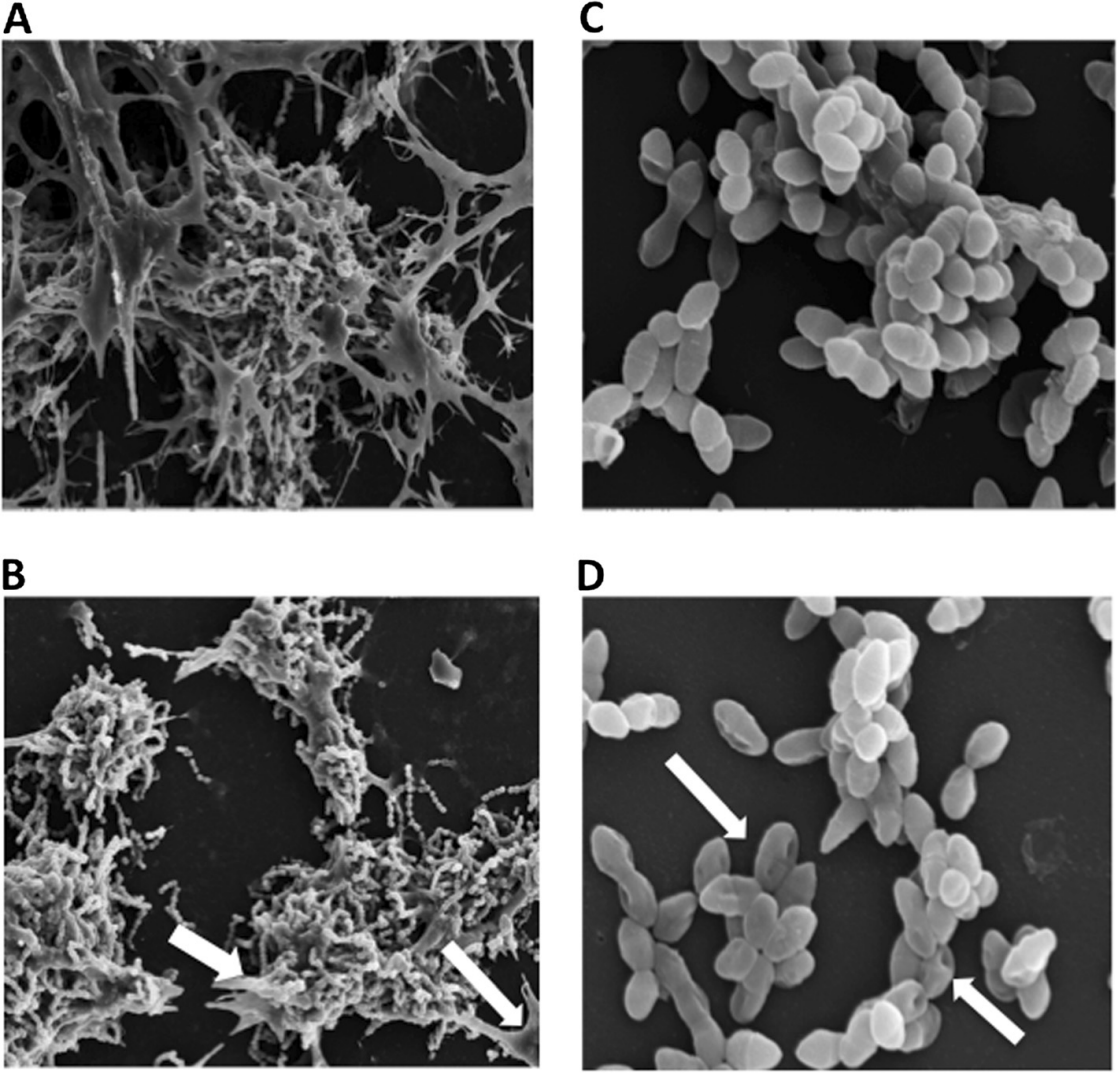
**CHD-FA reduces ECM and compromises cell membrane structure.***S. mutans* (x2000) and *E. faecalis* (x5000) biofilms were either untreated **(A** and **C,** respectively**)** or treated with 0.5% (v/v) CHD-FA **(B** and **D,** respectively**)** for 24 h on Thermanox™ coverslips. These were then processed and viewed on a JEOL JSM-6400 scanning electron microscope and images assembled using Photoshop software. Note the reduction in extracellular matrix **(B)** and perturbation of bacterial cell membranes **(D)**, denoted by arrows on sessile cells.

Further analysis of the impact of CHD-FA upon the physical cellular structure was assessed by SEM for representative biofilms. CHD-FA treatment reduced the overall quantity of *S. mutans* ECM (Figure 1B) and also caused perturbation of the cell membrane, as demonstrated by the punctured appearance of *E. faecalis* (Figure 1D).

### CHD-FA is effective against a multi-species periodontal biofilm

**Figure 2:**
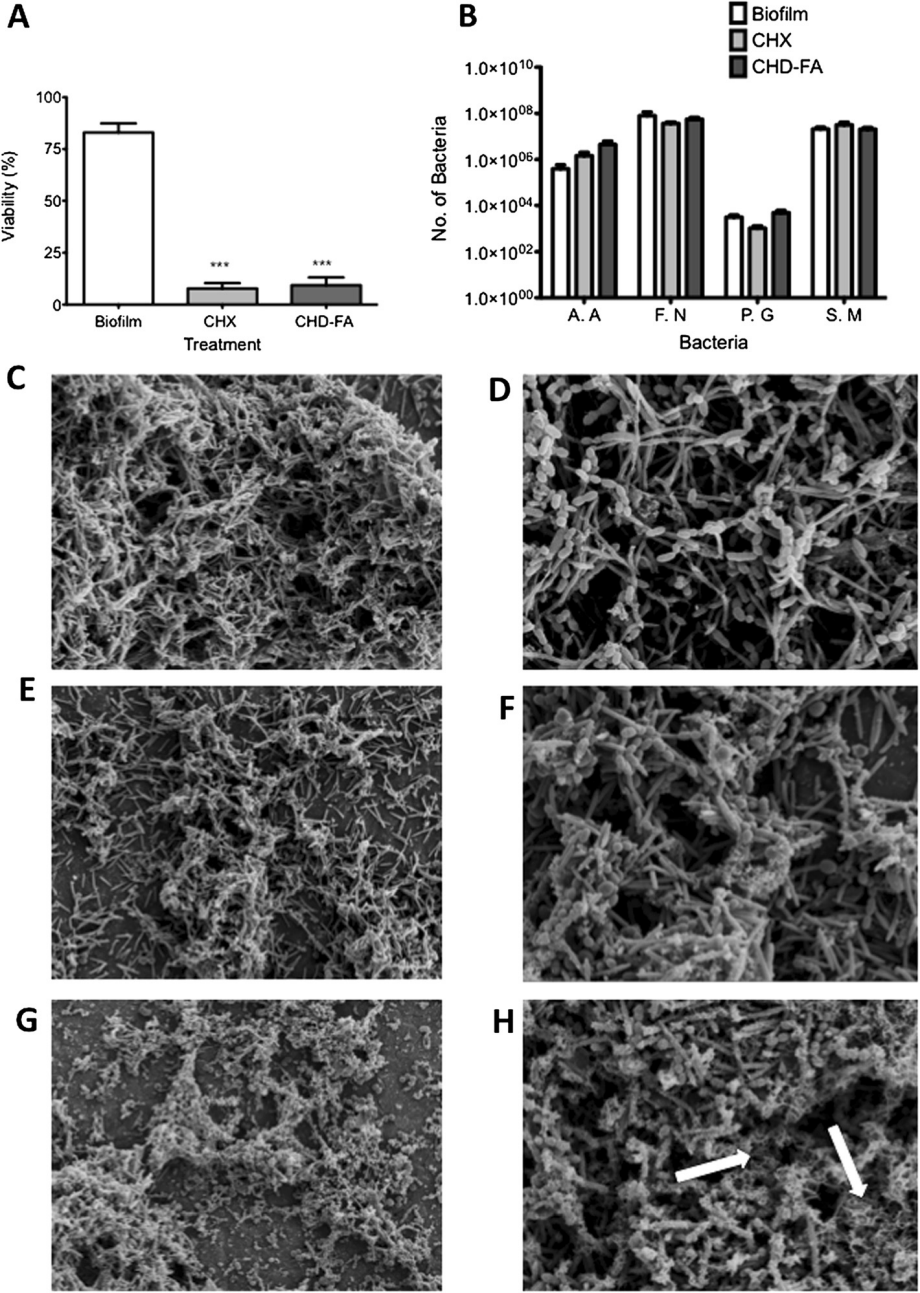
**CHD-FA kills and disrupts multi-species periodontal biofilms.** Multi-species periodontal biofilms were grown on Thermanox™ coverslips within 24 well plates for a total of 5 days, with AS media changed every day. Upon biofilm development, cells were treated with 0.5% (v/v) CHD-FA and 0.2% (v/v) CHX for 24 h before being washed with PBS. Reduction in metabolic activity was measured using the alamarBlue® assay **(A)**. All samples were assayed in triplicate, on three separate occasions. Data represents mean ± SD (***p < 0.0001). Biofilms were retained after treatment with CHD-FA or CHX and DNA was extracted for quantification of each species using SYBR® GreenER™ based qPCR **(B)**. Biofilms were also analysed by SEM at either 2000x **(C, E, G)** and 5000x **(D, F, H)**. These were processed and viewed on a JEOL JSM-6400 scanning electron microscope and images assembled using Photoshop software. Untreated multispecies biofilms were first compared at low magnification **(C)** to biofilms treated with 0.2% (v/v) CHX **(E)** and 0.5% (v/v) CHD-FA **(G)** for 24 h and it was shown that biofilm treatments caused disaggregation. At higher magnification the biofilms treated with 0.5% (v/v) CHD-FA for 24 h resulted in a fibrous ECM, as denoted by arrows **(H)**, as compared to the control **(D)** and CHX **(F)**.

Given that biofilms of the oral cavity are polymicrobial in nature, we developed a simple and reproducible multi-species model representative of sub-gingival plaque to test CHD-FA (Figure 2A). The antimicrobial activity of CHD-FA at 1 x SMIC was shown to significantly reduce cell viability to less than 10% (p < 0.0001), which was comparable to the CHX, which also significantly reduced cell viability to 8% (p < 0.0001). However, following treatment the number of each species within the biofilms were quantified and showed no significant reduction in biomass after CHD-FA or CHX treatment, compared to the untreated control (Figure 2B).

SEM analysis of these biofilms was then performed to evaluate any effect on the biofilm architecture (Figure 2C-H). At low magnification (x2000) both CHX and CHD-FA were shown to disrupt biofilm architecture (Figure 2E and G) when compared to the untreated control (Figure 2C), as shown by areas of sparse disaggregated biofilms. Moreover, at high magnification (x5000) CHD-FA also appeared to alter the overall physical appearance of the biofilm matrix with greater quantities of fibrous ECM observed as denoted by the arrows (Figure 2H), compared to the control and CHX (Figure 2D and F).

### CHD-FA alters the expression of pro-inflammatory mediators

**Figure 3:**
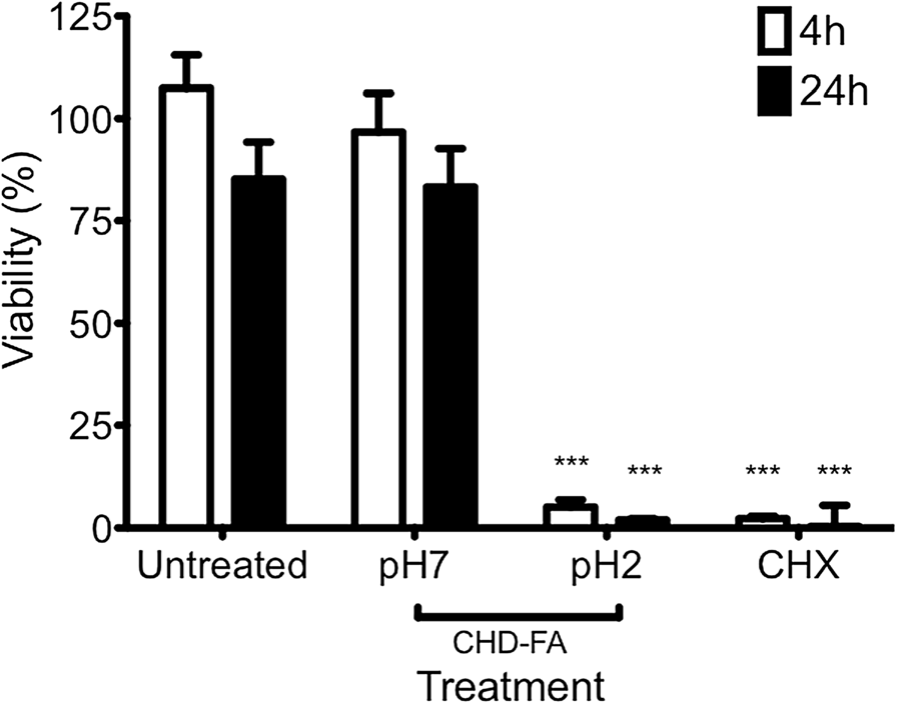
**CHD-FA is non-toxic against an oral epithelial cell line.** An orally relevant epithelial cell line (OKF6/TERT2) was grown to 90% confluence in 24 well plates for toxicity studies. Cells were treated with 0.5% (v/v) CHD-FA at pH 2.0 and 7.0 and with 0.2% CHX for 30 min. After treatment, cells were carefully washed with PBS and cellular viability assessed using the alamarBlue® assay, with absorbance read at 570 and 600 nm. All samples were assayed in triplicate, on three independent occasions. Data represents mean ± SD (***p <0.0001).

CHD-FA toxicity was assessed using an orally relevant epithelial cell line to determine whether there were any detrimental effects from the compounds tested prior to immunomodulatory investigations. Both CHX and CHD-FA, at its native pH of 2.0, were shown to be highly toxic towards to epithelial cells, reducing viability to less than 10% after 30 min exposure (Figure 3). However, when CHD-FA was buffered to a neutral pH of 7.0, no significant decrease in cell viability was observed.

**Figure 4:**
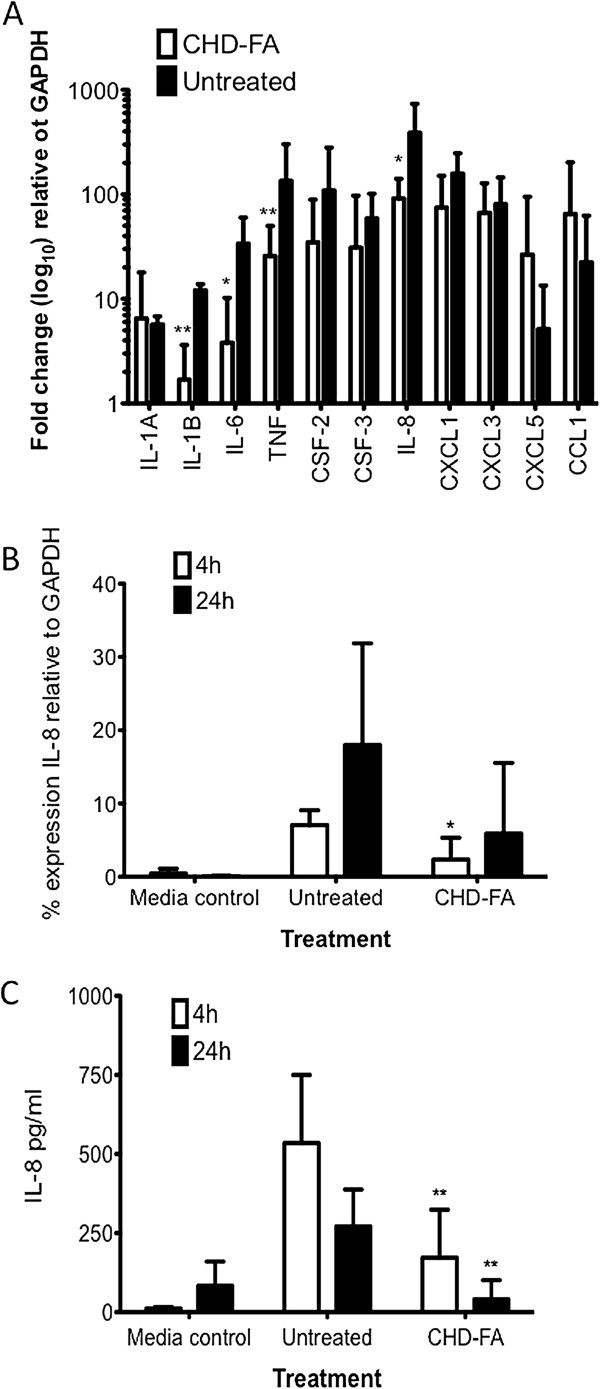
**CHD-FA modulates key inflammatory mediators *in vitro*.** An oral epithelial cell line (OKF6) was grown to 90% confluence in 24 well plates for assessing the effect of CHD-FA on the host immune response. Cells were pre-treated with 0.5% (v/v) CHD-FA pH 7.0 for 30 min, washed with PBS and stimulated with the multi-species periodontal biofilm for 4 and 24 h. Untreated controls were also included. Samples were assayed in triplicate and on three separate occasions. RNA was extracted from the 4 h cell lysates, cDNA synthesised and used in the RT^2^ Profiler to analyse the expression of a panel of pro-inflammatory mediators **(A)**. Duplicate samples from two independent experiments were used in the RT^2^ Profiler. Data is represented by mean + 95% CI, relative to untreated control. Samples were also assayed for IL-8 gene expression using SYBR® GreenER™ based qPCR **(B).** Expression of IL-8 is represented relative to housekeeping gene; GAPDH. Retained supernatants were also used to measure IL-8 protein release by ELISA **(C)**. All samples were assayed in triplicate, on three independent occasions. Data represents mean ± SD (*p < 0.05, **p < 0.01).

We next set out to determine whether or not CHD-FA was able to induce a biological response from the epithelial cells, principally by measuring changes in immune mediators. To evaluate this, a four-species biofilm model was developed, where no toxicity issues were observed when in contact with the epithelial cell line at 4 h (data not shown). Moreover, we have shown that CHD-FA treated cells do not significantly alter the release of IL-8 after 4 h and 24 h (data not shown). Initial gene expression studies using the RT^2^ Profiler on epithelial cells pre-treated with CHD-FA prior to biofilm challenge showed a general down-regulation of pro-inflammatory mediators (Figure 4A). Significant down-regulated genes included IL-6 (8.5 fold [p = 0.018]), IL-1β (7.05 fold [p = 0.012]), TNFα (5.22 fold [p = 0.013]) and IL-8 (4.24 fold [p = 0.021]).

We next focussed on IL-8 expression, one of the significantly affected genes and a key mediator of periodontal inflammation. At the mRNA level, IL-8 was significantly down-regulated in cells pre-treated with CHD-FA after 4 h, when compared to the untreated control (p = 0.0383) (Figure 4B). No statistically significant difference was observed after 24 h stimulation (p = 0.1712). In addition, at the protein level, IL-8 release was shown to be significantly down-regulated when cells were pre-treated with CHD-FA, after 4 h (p = 0.008) and 24 h (p = 0.0037) biofilm stimulation (Figure 4C). CHD-FA alone had no effect on oral epithelial cell IL-8 mRNA or protein expression (data not shown).

## Discussion

Oral microbial diseases are typically mediated by biofilms; communities of microorganisms that co-aggregate as sticky and tenacious structures and which characteristically have increased resistance to antimicrobials [25]. We recently reported that mouthwashes, including CHX were ineffective against a range of clinical MRSA strains [10], suggesting that alternative antimicrobial agents ought to be investigated. Indeed, recent studies in *C. albicans* have shown that the use of naturally derived molecules are effective against both orally and systemically derived isolates [14,15]. Here we report that CHD-FA, a naturally derived antiseptic molecule, displays rapid broad-spectrum antimicrobial activity, and also elicits immunomodulatory activity.

We first undertook a comparative assessment of CHD-FA and CHX against a range of important bacteria associated with oral biofilm infections. Both molecules were shown to effectively inhibit and kill planktonic cells, and both compounds were also effective against biofilms. Antimicrobial activity against planktonic bacteria has been reported previously for oxifulvic acid, a derivative of CHD-FA, where inhibition was observed against a range of important clinical pathogens, including *Pseudomonas aeruginosa, Staphylococcus aureus* and *Streptococcus pyogenes*[26]. Notably, only marginally higher concentrations of CHD-FA were required to kill the biofilm as compared to planktonic cells, whereas for CHX the fold change was up to 64 times, a phenomenon also reported for CHD-FA against *C. albicans* biofilms [15]. Moreover, CHD-FA showed a rapid rate of kill for the periodontal pathogens tested as polymicrobial biofilms, as after 30 min treatment cellular viability was reduced by ≥90%, which was also observed for studies of *C. albicans*[15]. It is recognised that a potential limitation of this study is that it was performed on biofilms produced from laboratory strains of the periodontal organisms *in vitro* and it is conceded that further investigation may be required to assess the anti-microbial properties against the most virulent of clinical strains and biofilms formed from *ex vivo* biofilms or *in vivo* within experimental gingivitis models. Nevertheless, collectively, these data suggest that CHD-FA has potent and broad-spectrum activity against microbial biofilms. Furthermore, the SEM images indicate an action against the bacterial cell membrane. Interestingly, the biofilms appeared to be disaggregated and displayed a fibrous appearance, presumably as a consequence of cell lysis and release of intracellular components of the bacterial cells. Our previous studies on *C. albicans* biofilms do not corroborate this observation, where no disruption of biofilms was observed [15]. However, the filamentous nature of *C. albicans* biofilms may explain why the compound was unable to disaggregate these. Despite this finding, there was no significant difference in the number of each species when treated with antimicrobial therapy, when compared to the untreated control. Though our assay was unable to determine whether these were live or dead.

Given that CHD-FA displayed an excellent antimicrobial profile, we wanted to ascertain whether it possesses any other biological properties, as has also been demonstrated for other naturals including tea tree oil [27]. It has been reported that CHD-FA has no toxicity in rats and humans, and it has been further suggested that it elicits anti-inflammatory and wound healing promoting properties [17,28]. Periodontitis is characterised by chronic inflammation that leads to tissue and bone destruction [29], therefore controlling these processes is an attractive option for clinical management. The use of *in vitro* multi-species oral biofilms to study the inflammatory processes driven by complex biofilms have been shown to be important [30], therefore we developed a similar system to test CHD-FA and other bioactive molecules. Both CHX and CHD-FA in their native forms were shown to be toxic, therefore, in order to demonstrate subtle biological effects we buffered CHD-FA to pH 7.0 in order to test our hypothesis that it was immunomodulatory *in vitro*. Using an orally relevant epithelial cell line stimulated with a polymicrobial biofilm we demonstrated that at the transcript level cells treated with CHD-FA showed a significant down-regulation of pro-inflammatory molecules, including the chemokine IL-8. Analysis of the IL-8 protein also showed a significant reduction in its release from oral epithelial cells. These data indicate that CHD-FA has bioactivity against mammalian cells, as has been reported elsewhere [17,28]. However, we accept these differences are only observed when CHD-FA is adjusted to a neutral pH, therefore, further studies are required to determine the most suitable formulation of CHD-FA to potentially be used clinically, which at present is formulated at pH 2.8. At present, however, the precise mechanism of action remains unknown, but we can only speculate that CHD-FA interacts with membrane proteins resulting in blocking signalling pathways, which leads to down-regulation of pro-inflammatory mediators on stimulation with biofilms. This is currently subject to further investigation by our group.

## Conclusions

This study has demonstrated that the naturally derived compound CHD-FA exhibits broad-spectrum antimicrobial activity against orally relevant biofilm organisms. Although a four species mixed biofilm model was used in this study, we are aware that antimicrobial activity against this model does not fully represent all mixed biofilms that are found within the oral cavity, but only a few of species relevant in periodontal disease. It further shows that CHD-FA has the capacity to modulate the immune response and down-regulate the biofilm induced expression of pro-inflammatory mediators in oral keratinocytes. However, a further limitation of this study was only a selected number of inflammatory mediators were investigated, thus precluding other host factors for consideration, which may influence the inflammatory response even further. Collectively, these properties make CHD-FA an attractive option for the development of a mouthwash to treat microbial oral disease; although further studies *in vitro* and *in vivo* are first required to further define the mode of action of this unique compound.

## Abbreviations

CHD-FA: Carbohydrate derived fulvic acid; ECM: Extracellular matrix; CHX: Chlorhexidine; SEM: Scanning electron microscopy; CI: Confidence intervals; SD: Standard deviation; PMIC: Planktonic minimum inhibitory concentration; PMBC: Planktonic minimum bactericidal concentration; SMIC: Sessile minimum inhibitory concentration; TTO: Tea tree oil; MRSA: Methicillin resistance *Staphylococcus aureus.*

## Competing interests

The PhD studentship stipend of LS was partially funded by Fulhold Ltd, who also provided CHD-FA for the experimental procedures.

## Authors’ contributions

LS participated in the study design, carried out the experimental studies, performed statistical analysis and was responsible for the manuscript. EM produced and ran the multi-species biofilm model. DFL participated in study design, assisted with statistical support and helped draft the manuscript. CM participated in study design and supervised manuscript writing. SC contributed to study design, data analysis and supervised manuscript writing. CJN contributed to the immunological study design, data analysis and contributed to the manuscript writing. GR conceived the study, participated in study design and was jointly responsible for writing the final manuscript. All authors read and approved the manuscript.

## Pre-publication history

The pre-publication history for this paper can be accessed here:

http://www.biomedcentral.com/1472-6831/13/47/prepub
